# Chronic Treatment with Oxcarbazepine Attenuates Its Anticonvulsant Effect in the Maximal Electroshock Model in Mice

**DOI:** 10.3390/ijms25126751

**Published:** 2024-06-19

**Authors:** Kinga Borowicz-Reutt, Monika Banach

**Affiliations:** Independent Unit of Experimental Neuropathophysiology, Department of Toxicology, Medical University of Lublin, Jaczewskiego 8b, 20-090 Lublin, Poland; monikabanach@umlub.pl

**Keywords:** oxcarbazepine, antiepileptic drugs, chronic treatment, epilepsy, maximal electroshock-induced seizures

## Abstract

The objective of this study was to assess the impact of acute and chronic treatment with oxcarbazepine on its anticonvulsant activity, neurological adverse effects, and protective index in mice. Oxcarbazepine was administered in four protocols: once or twice daily for one week (7 × 1 or 7 × 2) and once or twice daily for two weeks (14 × 1 or 14 × 2). A single dose of the drug was employed as a control. The anticonvulsant effect was evaluated in the maximal electroshock test in mice. Motor and long-term memory impairment were assessed using the chimney test and the passive avoidance task, respectively. The concentrations of oxcarbazepine in the brain and plasma were determined via high-performance liquid chromatography. Two weeks of oxcarbazepine treatment resulted in a significant reduction in the anticonvulsant (in the 14 × 1; 14 × 2 protocols) and neurotoxic (in the 14 × 2 schedule) effects of this drug. In contrast, the protective index for oxcarbazepine in the 14 × 2 protocol was found to be lower than that calculated for the control. No significant deficits in memory or motor coordination were observed following repeated administration of oxcarbazepine. The plasma and brain concentrations of this anticonvulsant were found to be significantly higher in the one-week protocols. Chronic treatment with oxcarbazepine may result in the development of tolerance to its anticonvulsant and neurotoxic effects, which appears to be dependent on pharmacodynamic mechanisms.

## 1. Introduction

Chronic diseases such as epilepsy necessitate long-term treatment, which can be complicated by the occurrence of serious adverse drug reactions. Despite the administration of optimal therapy, it is often unfeasible to prevent the disease from progressing. Initially, a single antiepileptic drug is employed, and when it proves ineffective, another drug is utilized instead of or in conjunction with the initial drug. Both monotherapy and polytherapy have their respective advantages and disadvantages. Patients are more likely to adhere to a straightforward drug regimen than a complex one, which may result in poor compliance. Single-drug therapy offers a superior benefit–risk ratio in comparison with polytherapy, which is more frequently associated with adverse effects or drug–drug interactions. Finally, the cost of monotherapy is typically lower. 

The present study concerns the chronic administration of oxcarbazepine (OXC). OXC is a 10-keto analog of carbamazepine designed to improve the side effect profile. Concurrently, OXC preserves the efficacy of the parent drug. The primary distinction between the two drugs is that OXC is not metabolized to an epoxide derivative, which is responsible for the toxic effects of carbamazepine [1,2].

The efficacy and safety of OXC were evaluated in 67 clinical trials for epilepsy, peripheral neuropathic pain, bipolar disorder, multiple sclerosis, and even bronchial asthma [3]. As recently as eight years ago, OXC was considered the drug of first choice in the treatment of partial seizures [4]. Currently, OXC is recommended as a second-line monotherapy or adjunctive treatment for focal-onset seizures with or without secondary generalization in both children and adults [5]. Another indication for OXC is trigeminal neuralgia [6].

Although levetiracetam is currently the most commonly used drug for focal epilepsy, some studies have demonstrated that treatment with OXC resulted in significantly higher rates of seizure freedom and 12-month retention. Furthermore, OXC demonstrated superior efficacy in pediatric patients with self-limited infantile epilepsy and non-syndromic epilepsy of genetic or unknown etiology. Furthermore, OXC monotherapy demonstrated superior efficacy compared with levetiracetam in the treatment of benign epilepsy with centrotemporal spikes. The incidence of adverse events was comparable between OXC and levetiracetam. Nevertheless, children treated with OXC exhibited greater improvements in intelligence indices and cognitive functions. Consequently, OXC can be considered a suitable first-line therapy for the aforementioned cases of pediatric epilepsy, as evidenced by the findings of Zhao et al. [7] and Suo et al. [8]. Furthermore, OXC can be employed in instances where levetiracetam is contraindicated, such as in cases of psychiatric disorders or hypersensitivity to LEV.

In animal studies, OXC demonstrated anticonvulsant efficacy in a maximal electro-shock model in mice and in amygdala kindling and pilocarpine-induced status epilepticus in rats [9]. It is noteworthy that the majority of experiments were conducted using an acute protocol, which entailed administering a single dose of the drug. In clinical settings, individuals diagnosed with epilepsy require chronic, even lifelong, treatment. The efficacy and safety of pharmacotherapy may undergo changes over time during long-term therapy. This is due to pharmacokinetic and/or pharmacodynamic events. Consequently, it appears prudent to compare the efficacy of antiepileptic drugs in acute and chronic protocols in primary preclinical screening tests.

Prior to its approval as an antiepileptic drug, OXC underwent extensive preclinical and clinical testing. The objective was not to expand the existing knowledge base regarding the properties of OXC. This study is part of a larger project, which aims to evaluate whether the single application of antiepileptic drugs commonly used in experimental research is equivalent to their chronic administration. If the results are not reliable, then studies should be conducted chronically, mimicking clinical conditions, in order to increase the reliability of the results. It is hoped that the implementation of this rule will result in a reduction in the number of unnecessary animal experiments, which is highly desirable from an ethical standpoint.

The present study evaluated the effects of repeated administration of OXC on its anticonvulsant activity in the maximal electroshock (MES) test, neurological side effects, protective index, and plasma and brain concentrations in mice.

## 2. Results

### 2.1. Maximal Electroshock Test

**Figure 1:**
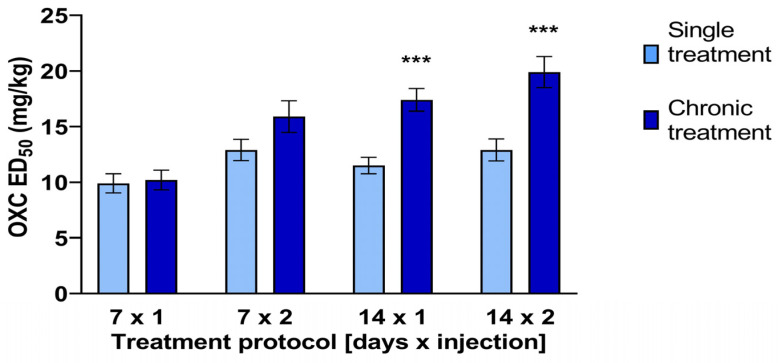
The effects of acute and chronic treatment with oxcarbazepine (OXC) on its antielectroshock activity mice. The data are presented as median effective doses (ED_50_ with SEM values), which indicate the dose required to protect 50% of the animals against seizures. OXC was administered acutely or chronically (in four protocols) 30 min prior to testing. The following treatment protocols were employed: 7 × 1, 1 injection daily for 7 days (the control group); 7 × 2, 2 injections daily for 7 days; 14 × 1, 1 injection daily for 14 days; 14 × 2, 2 injections daily for 14 days. The results demonstrated a significant reduction in seizure frequency compared with that in the control group (single administration of OXC), with *** *p*-values < 0.001.

The antielectroshock effect of OXC exhibited temporal variability contingent upon the drug administration regimen. The ED_50_ (50% effective dose) of OXC administered in the 14 × 2 protocol was found to be significantly higher than the control value (the 1 × 1 protocol). The ED_50_2 (19.9 ± 1.4 mg/kg) was found to be significantly higher than the ED_50_1 (12.9 ± 0.99 mg/kg) (t(62) = 3.561, *p* = 0.0007). Similarly, the ED_50_ of OXC administered in the 14 × 1 protocol was significantly higher than the control value: 17.4 ± 1.02 mg/kg vs. 11.5 ± 0.74 mg/kg (t(44) = 3.926; *p* = 0.0003). No significant differences were observed in the one-week dosing protocols. The attenuation of the anticonvulsant effect in the chronic (14 × 1 and 14 × 2) protocols did not appear to be due to pharmacokinetic interactions, as plasma and brain drug concentrations did not differ significantly from control values (see also Section 2.4). To exclude the influence of seasonal rhythms on the ED_50_ value, each chronic protocol was followed by a new control. Figure 1 depicts the impact of acute and chronic administration of OXC on maximal electroshock-induced seizures.

### 2.2. Chimney Test

The administration of OXC, whether acute or chronic, at doses of 12.9 mg/kg (ED_50_1) and 19.9 mg/kg (ED_50_2), did not affect motor coordination in mice. All animals exited the chimney tube within a few seconds (Table 1). Given that no impairment was observed in mice receiving the highest total dose of OXC, it was deemed inappropriate to proceed with the chimney test in the remaining protocols.

TD_50_ values after acute and chronic (14 × 2) administration of OXC were determined in the chimney test. The median toxic dose of OXC after single injection was 66.4 ± 2.16 mg/kg. In the chronic protocol, OXC toxicity was attenuated as the TD_50_ was 89.6 ± 5.78 mg/kg. The TD_50_ value in the chronic protocols was significantly higher compared with that in the acute experiment (t(80) = 4.338, *p* < 0.0001) (Table 2). Consequently, the protective index of chronic OXC (4.5) was lower than that assessed after the acute treatment with OXC (5.15) (Table 2).

### 2.3. Step-Through Passive Avoidance Test

The results of the step-through passive avoidance task in mice indicated that there was no significant deficit in long-term memory following single and repeated (14 × 2) administration of OXC at the two doses of ED_50_1 and ED_50_2 (Table 3).

### 2.4. Brain and Plasma Concentrations of OXC

**Figure 2:**
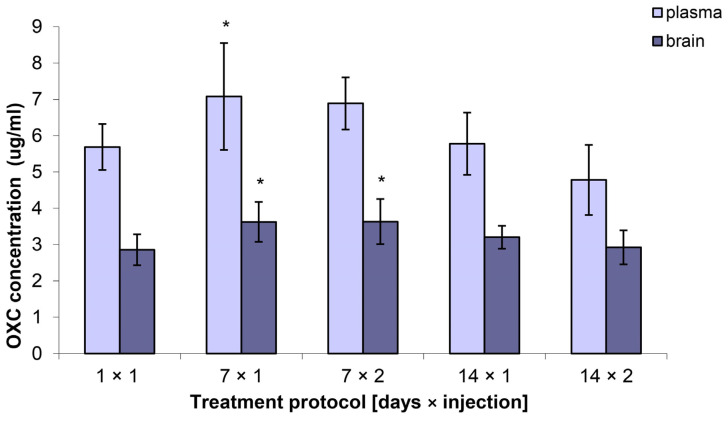
Plasma and brain concentrations of OXC following acute and chronic treatment. The results are presented as the mean ± SD of at least eight determinations. A one-way analysis of variance (ANOVA) was employed to analyze the plasma and brain concentrations of OXC, followed by a post hoc Dunnett test. A single dose of OXC was employed as a control. OXC—oxcarbazepine. The experimental groups were as follows: 7 × 1, 1 injection daily for 7 days; 7 × 2, 2 injections daily for 7 days; 14 × 1, 1 injection daily for 14 days; 14 × 2, 2 injections daily for 14 days. The control group received a single injection of OXC. The results were statistically significant at * *p* < 0.05.

The plasma concentration of OXC (MHD) as measured in the subchronic protocol (7 × 1) was found to be significantly higher when compared with that in the control group (a single drug injection). The plasma concentrations of OXC, as measured in the remaining protocols (7 × 2, 14 × 1, and 14 × 2), were not found to be significantly different from those in the control. The brain concentrations of OXC were found to be significantly higher in the subchronic groups (7 × 1 and 7 × 2) when compared with those in the control group. In both two-week chronic protocols, the brain OXC concentrations did not differ from the control (Figure 2).

## 3. Discussion

The MES test in mice is a well-validated preclinical model of tonic–clonic seizures in humans. The procedure is designed to minimize distress to the animal by limiting the duration of the seizure to 0.2 s, which is the typical time frame for the current application. Anticonvulsant drugs that block sodium channels (such as OXC) have been demonstrated to be particularly effective against the MES test [9,10,11]. The results of our study indicate that chronic treatment with OXC attenuates its anticonvulsant effect in the MES test in mice. The ED_50_ values of OXC were found to be significantly higher than those of the control ED_50_s in the context of chronic protocols. The ED_50_ values of this anticonvulsant administered in subchronic protocols were not statistically different from the control. 

The available literature does not provide examples of the development of tolerance to the anticonvulsant effects of OXC. A limited number of articles address the chronic administration of the drug. The administration of OXC and its monohydroxymetabolite (MHD) once daily per os for 4 weeks in the dose range of 6–30 mg/kg did not result in any alterations to their antielectroshock effects in rats. Chronic treatment with OXC and MHD (up to 100 mg/kg per os for 25 days) prior to electrical stimulation did not affect the process of kindling in rats. This indicated that OXC had no effect on the process of epileptogenesis. In contrast, acute oral administration of OXC and MHD demonstrated anticonvulsant properties in mice and rats, with ED_50_ values of 13.5 and 20.5 mg/kg, respectively. Additionally, a single dose of OXC (7.5 mg/kg) was found to inhibit seizures in amygdala-kindled rats [9,12,13]. Similarly, both acute and chronic (once daily for 4 weeks) treatment with OXC at different dose ranges and routes (50–100 mg/kg per os or 20–60 mg/kg i.m.) reduced or abolished aluminum-induced partial seizures in rhesus monkeys [12]. The results of our study indicate that OXC has anticonvulsant effects following both single and repeated administration. A partial difference was observed between subchronic and chronic administration. In light of the findings from the aforementioned studies, which examined the effects of once-daily chronic administration, it can be concluded that the impact of OXC in the 7 × 1 protocol was comparable to that observed following a single injection. In the 14 × 1 protocol, the antiepileptic efficacy was reduced but not abolished in comparison with the acute application. The discrepancy between our studies and those mentioned above [9,12,13] may be attributed to the use of different rodent species (rats vs. mice) and routes of drug administration.

The plasma and brain concentrations of OXC in the chronic groups were not found to differ from the controlled values. Consequently, it can be concluded that pharmacokinetic events are not responsible for this effect. Furthermore, we postulated that pharmacodynamic tolerance may be a potential explanation for the observed findings.

Two main mechanisms of tolerance development have been identified. Pharmacokinetic tolerance is the result of the induction of liver enzymes that metabolize AEDs, which subsequently leads to a reduction in drug plasma and brain concentrations. This can be circumvented by increasing the dosage of the drug in question. In the present study, we excluded pure pharmacokinetic events. Conversely, there is also the phenomenon of pharmacodynamic tolerance, which arises due to the adaptive downregulation of receptors and other molecular target points for AEDs. Conversely, this phenomenon is reversible upon cessation of the drug [14]. The results of this study indicate that the tolerance observed in mice is likely to have a pharmacodynamic mechanism. 

In preclinical studies, the degree of tolerance exhibited by subjects varied depending on the specific seizure model, the type of drug administered, and the dosage employed. Furthermore, a post hoc analysis of several randomized clinical trials revealed that 14% of patients exhibited tolerance to OXC. The occurrence of complete remission of seizures during OXC monotherapy or a ≥50% reduction in symptoms for adjunctive therapy was considered a satisfactory outcome at 3 and 6 months of treatment. In this time frame, 15% of patients with drug-resistant epilepsy who were treated with OXC achieved satisfactory seizure control. A loss of antiseizure effect was observed in 5% of patients undergoing monotherapy and 9% undergoing polytherapy. In the placebo group, tolerance to the antiseizure effect was observed in 4% of individuals. This suggests that tolerance to OXC is relatively low. Further observation of the same population after 6 and 12 months of OXC therapy revealed that only 10% of patients with adequate seizure control at 6 months did not maintain this state at 12 months. Moreover, only 4% of patients who were seizure-free at 6 months experienced two or more seizure episodes during the subsequent 6 months. The observed reduction in anticonvulsant effect may be attributed to the development of tolerance to OXC. Nevertheless, it can be posited that the response observed at 6 months is a reliable predictor of the response observed at 12 months [15]. In a separate study, the long-term effects of 6-month OXC monotherapy were evaluated in a prospective cohort study involving 167 patients diagnosed with focal epilepsy. The retention rate for this anticonvulsant at the third year of treatment was 41.8%, which was a superior result to that observed for carbamazepine or valproate. Finally, the seizure remission rate was 75.3% [16]. Upon comprehensive analysis of the presented data, it was found that long-term treatment with OXC resulted in a loss of antiseizure effect in 5 to 14% of patients, while 15 to 45% of patients achieved better seizure control. However, the improvement was not unexpected, as it had been observed in newly diagnosed patients [14,15]. Nevertheless, despite the methodological differences between the studies discussed above, the results suggest that the development rate of tolerance to OXC is relatively low. 

The subsequent phase of our study involved the assessment of motor impairment and long-term memory deficits in mice in the classic chimney test and passive avoidance task, respectively. Neither a significant deficit in memory nor motor impairment was observed following the acute or repeated administration of OXC. Nevertheless, the motor neurotoxicity induced by chronic OXC (TD_50_ dose) was significantly lower than that observed in the control group. In detail, the calculated TD_50_ value for the anticonvulsant in the chronic protocol was 35% higher than that in the control group. In conclusion, the results of the classic chimney test indicate that chronic treatment with OXC does not result in significant neurotoxic effects in mice. In humans, OXC may induce some motor disorders, such as dystonia, myoclonus, and Parkinsonism. However, the symptoms observed in mice in our study did not resemble such disturbances [17,18].

To date, there is no published literature on the toxic dose (TD_50_) of OXC when administered chronically. The values assessed following a single administration are as follows: The results of the chimney test in mice indicated a dose of 67.5 mg/kg, while the rotarod test yielded a value of 45.7 mg/kg [19,20]. The results were found to be practically identical, with a TD_50_ of 66.4 mg/kg. The protection index for acute OXC-induced motor impairment, calculated for OXC by Schmutz et al. [13], was 10. The same parameter in our study was 5.15; however, in the cited study, motor impairment was evaluated in the rotarod test, whereas in our study it was evaluated in the chimney test. Therefore, the observed discrepancy may be attributed to methodological differences. The lethal dose (LD_50_) of OXC in mice is not known. Conversely, the LD_50_ of this drug in mammals after oral administration was determined to be 1240 mg/kg [21].

The process of epileptogenesis may impair cognition in animals and humans. Moreover, the administration of antiepileptic drugs is thought to exacerbate this issue. Agarval et al. [22] evaluated long-term memory in non-epileptic and PTZ-kindled mice following chronic administration of OXC (15 mg/kg per os for 5 weeks). Neither control nor epileptic animals exhibited significant memory deficits in the elevated plus maze test and passive avoidance task when compared with vehicle-treated mice. Furthermore, no significant impact of chronic OXC treatment on the concentration of oxidative stress markers (malondialdehyde, glutathione, superoxide dismutase, or catalase activity) was observed [22]. The results of this study confirm that OXC does not induce memory impairment in mice [18].

As previously established, OXC produces fewer undesired effects than carbamazepine. The most common adverse effects are headache, dizziness, ataxia, weakness, nausea, vomiting, and hyponatremia, particularly in the elderly [23]. As reported by Grant and Faulds [24], the replacement of CBZ with OXC has been shown to improve cognitive function in epileptic patients. In a study involving newly diagnosed patients with focal epilepsy, monotherapy with OXC for 232 days (approximately eight months) demonstrated improvements in verbal and visual memory, attention, and working memory [25]. In two additional studies, neuropsychological tests were conducted in patients with newly diagnosed epilepsy who had been treated with OXC for 4 to 12 months. No impairment of cognitive function was reported in the short- to medium-term observations [26,27]. Moreover, in patients with refractory medial temporal lobe epilepsy related to hippocampal sclerosis, OXC did not impair general cognition, working memory, episodic memory, executive functions, and language abilities. The sole parameter to exhibit a decline was visual denomination [28]. In general, OXC monotherapy does not appear to have a significant impact on cognitive functions in humans. It is noteworthy that OXC augmented the favorable effects of a three-month course of LEV treatment on memory and cognition in patients with temporal lobe epilepsy. The observation group (treated concomitantly with LEV and OXC) demonstrated superior performance on measures of declarative memory and intellectual acuity relative to the control group treated with levetiracetam monotherapy. The authors posit that this effect may be attributed to the inhibition of calcium ion efflux and the promotion of potassium ion influx, which, over time, stimulates neurogenesis and synaptogenesis [29]. 

The results of our study indicate that chronic administration of OXC results in the development of tolerance to both antielectroshock and the motor neurotoxic effects in mice. It is likely that these effects are mediated by similar or identical mechanisms. However, the increase in the ED_50_ value was greater than that in the TD_50_ value (54% vs. 35%), indicating that tolerance to anticonvulsant effects is also greater compared with that to neurotoxic action. Consequently, the protective index of OXC following chronic treatment (14 × 2) was found to be diminished in comparison with that of the control.

It is possible that an understanding of the pharmacokinetic profile of OXC may assist in explaining the results obtained. It is important to note that this profile is specific to humans. The pharmacokinetics of OXC in mice remains to be elucidated. In humans, OXC is a prodrug with bioavailability reaching 95–100%. It is rapidly absorbed from the gastrointestinal tract and converted into the 10-monohydroxy derivative (MHD). Subsequently, it is glucuronidated. The enzymes involved in the metabolism of OXC (ketoreductase and UDP-glucuronyltransferase) are not inducible. The elimination of OXC as an MHD or glucuronide occurs via the kidney. Only 4% of MHD is oxidized to the inactive 10,11-trans-dihydroxycarbamazepine. OXC is a lipophilic particle that is insoluble in water. Both OXC and MHD are readily transported across the blood–brain barrier. In comparison with OXC, MHD is less likely to bind plasma proteins, with a binding ratio of 40% vs. 68% [1,4,30,31,32]. The maximal serum concentration of OXC can be achieved after 3–6 h, with a stationary status being reached after 2–3 days [32,33,34,35]. The maximal serum concentration of MHD after oral administration can be achieved after 4–12 h, and at the stationary status after 2–4 h. The calculated half-life of OXC is 1–5 h, whereas that for MHD is 7–20 h. The therapeutic concentration of OXC and MHD oscillates between 3–35 and 10–35 µg/mL, depending on the author [30,35,36,37]. In our study conducted on mice, the plasma concentration of this anticonvulsant exhibited a slight decline, from 5.80 µg/mL to 4.66 µg/mL, in the 14 × 1 and 14 × 2 protocols, respectively. OXC is available in tablet and oral suspension forms. In humans, the dosage is 150–1200 mg per 24 h, with a maximum dosage of 2400 mg per 24 h [38,39,40]. A method of dose translation from animals to humans is available, utilizing the body surface normalization method. Calculation of the ED_50_ values for OXC in the mouse MES test (12.9 mg/kg in the 1 × 1 protocol and 19.9 mg/kg in the 14:1 schedule) yielded values of 1.05 and 1.61 mg/kg, respectively [41]. This suggests that mice require a higher dose of OXC to inhibit seizures, likely due to the faster elimination of the drug in rodents. Consequently, any correlation between ED_50_ values in mice and human therapeutic doses should be discussed in conjunction with the plasma therapeutic concentration [42,43,44,45].

Continuing the above thread, the total brain concentration of OXC in our study was significantly higher than that in the control in the two subchronic protocols (7 × 1, 7 × 2). Meanwhile, the plasma concentration was higher than that in the control only in protocol 7 × 1. It appears that during repeated administration of OXC, its concentration first increased in the plasma, then the OXC passed to the brain, and later its cerebral concentration returned to control values. However, these changes were not reflected in the antielectroshock effect of OXC. In a similar manner, in the 2-week protocols, the ED_50_ and TD_50_ values exhibited a significant increase, without any change in the brain or plasma concentrations of OXC. This further supports the hypothesis that tolerance to antiseizure action or to the motor toxic effect cannot be solely attributed to metabolic mechanisms. Rather, its development is influenced by pharmacodynamic factors.

OXC does not induce the enzymes involved in its own metabolism. Nevertheless, OXC and MHD, particularly at higher doses, may influence the action of cytochromes employed in the metabolism of other drugs, including CBZ. Specifically, OXC and MHC inhibit CYP2C19, and weakly induce CYP3A4 and CYP3A5. In a separate study, the capacity of OXC to stimulate microsomal enzymes was evaluated using phenazone kinetics as a marker of induction. In a study involving healthy volunteers, the repeated administration of OXC at a dose of 600 mg/24 h for 15 days did not result in any changes to the pharmacokinetic parameters of phenazone, indicating that OXC does not affect the activity of CYP enzymes. Conversely, higher doses of OXC (1800 mg/24 h) resulted in a moderate (20%) reduction in the elimination half-life of phenazone, without affecting other pharmacokinetic parameters [33].

It has been demonstrated that there are species-specific differences in the metabolism of OXC in humans, rats, and dogs. In rats, the process of OXC reduction to MHD appears to be less significant than in humans. The elimination of OXC from the bloodstream is a gradual process, which results in a sustained concentration of the drug. Subsequently, OXC undergoes hydroxylation, which is inducible. The administration of OXC for a period of four days resulted in a significant induction of hepatic microsomal enzymes in rats. The effect of MHD in the same experiment was moderate and less pronounced than that of OXC [30,45]. To date, there are no literature data dealing with OXC metabolism in mice. Based on the results of this study, it can be hypothesized that OXC metabolism in mice is induced in a manner similar to that observed in rats. In our study, we observed a negligible reduction in plasma and brain OXC concentrations in the chronic versus subchronic protocols. It is possible that this may contribute to the development of tolerance to anticonvulsive and motor neurotoxic effects during chronic treatment in mice.

In order to assess the potential pharmacodynamic mechanisms for reducing the anticonvulsant and toxic properties of OXC, it is necessary to consider all membrane targets of this drug. However, the precise definition of these targets remains elusive. It is well documented that OXC and MHD bind to voltage-sensitive sodium channels in their inactive state, thus inhibiting the propagation of impulses and, in consequence, seizure spread. In addition to these antiseizure mechanisms, the two drugs have been shown to potentiate potassium conductance and regulate the activity of high-voltage activated N/P and R calcium channels, thereby decreasing neuronal excitability and synaptic transmission. To a limited extent, the two anticonvulsants block adenosine receptor A1, reduce presynaptic glutamatergic transmission [1,17,32,37], and increase serotonin and dopamine concentration in the hippocampus. Nevertheless, in vitro studies have demonstrated the inhibition of glutamatergic activity, although this effect has not been replicated in vivo [32,46,47,48]. The available literature does not contain any data on the mechanisms of tolerance development during chronic treatment with OXC. To the extent that we can extrapolate data on the development of CBZ resistance in kindled rats to the mechanisms of tolerance to OXC in mice, it can be postulated that decreased inhibition of sodium channels, especially shortened recovery from the inactivation state of fast sodium channel currents, may be a contributing factor [49].

Summing up, the effectiveness of pharmacotherapy may change during prolonged exposure to a given drug. Usually, both metabolic and functional mechanisms of this phenomenon are taken into consideration. Antiepileptic drugs may induce or inhibit liver enzymes, enhancing their autoinduction and/or metabolism of other drugs. However, this is not the case of OXC, which does not affect its own metabolism. Another implication of long-term drug treatment is the development of various adaptive responses, including modulation of receptor sensitivity and density. This option is mostly taken into account to explain the reduction in OXC antiseizure activity and motor neurotoxicity observed in our study. Nevertheless, the detailed mechanism is not known at this stage. 

A substantial proportion of studies that assess the efficacy of antiepileptic drugs in preventing seizures and their potential to interact with other medications are based on a single administration of the test substances. The results of the present study indicate that conclusions derived from acute experiments should be validated in prolonged protocols. It is also important to note that the extrapolation of effects observed in non-epileptic animals to those in epileptic animals is not straightforward. Consequently, the results of this study should be further validated in chronic seizure models and/or genetically altered mouse models of seizures. Finally, chronic treatment should be included in preclinical screening tests searching for new anticonvulsant drugs and in the assessment of interactions between antiepileptic drugs.

One of the most notable limitations of this study was the seizure model employed. On the one hand, the maximal electroshock is a straightforward screening test employed in preclinical studies. Conversely, experiments are conducted in naive animals (i.e., those not previously induced to have seizures), and the methodology of this test restricts experimentation with drug doses. At a certain dose, 100% protection against convulsions is consistently observed. Moreover, the results of experimental studies cannot be directly translated into clinical conditions. Our findings indicate that in animal studies, drugs should be administered chronically, rather than in a single injection. Among the potential avenues for further research, we can suggest the expansion of studies to include chronic seizure models in animals, such as kindled seizures in rats. In subsequent studies, greater emphasis will be placed on pharmacokinetic issues. It would be beneficial to correlate drug concentrations with the intensity of the anticonvulsant effect. Furthermore, we have initiated a project with the objective of elucidating the extent to which alterations in the anticonvulsant effect during chronic drug administration may be attributed to changes in the expression of selected genes.

## 4. Materials and Methods

### 4.1. Animals

The study was conducted on adult male Swiss mice (20–26 g). The animals were housed in groups of 10 in cages of appropriate size with ad libitum access to food and tap water. The animals were maintained under standardized laboratory conditions, including a natural light–dark cycle of 12 h–12 h, a temperature range of 20–24 °C, an air humidity range of 45–65%, and an air exchange rate of 15/h. Following a week of acclimatization, the mice were randomly assigned to one of the experimental groups, each containing eight to ten mice. All experiments were conducted between 9:00 a.m. and 2:00 p.m. Each mouse was utilized only once. All procedures were approved by the Local Ethical Committee (license numbers 35/2009 and 9/2012) and were conducted in accordance with European Union Directive 2010/63/EU for animal experimentation and the ARRIVE guidelines. 

### 4.2. Drug 

The study employed oxcarbazepine (OXC), also known as Trileptal, which is manufactured by Novartis Pharma GmbH in Nurnberg, Germany. The inactive ingredients present in Trileptal tablets include colloidal silicon dioxide, crospovidone, hydroxypropyl methylcellulose, iron oxide, magnesium stearate, microcrystalline cellulose, polyethylene glycol, talc, and titanium dioxide. The tablets were crushed and suspended in an aqueous solution of Tween 80. Each day, a fresh suspension was prepared. The antiepileptic drug was administered intraperitoneally (i.p.) in a volume of 0.01 mL/g body weight. The duration of drug administration in chronic protocols was determined based on data from the literature [50,51]. Four treatment protocols were employed for repeated administration of OXC.

1. A single injection was administered every 24 h for seven days (7 × 1 subchronic treatment);
2. Two injections were administered daily, with a 12 h interval between doses, for seven days (7 × 2 subchronic treatment);
3. A single injection was administered every 24 h for 14 days (14 × 1 chronic treatment);
4. Two injections were administered daily, with a 12 h interval between doses, for 14 days (14 × 2 chronic treatment).

Additionally, four control groups were included (1 × 1 acute treatment), one for each chronic protocol. The control animals were administered a vehicle in accordance with the respective chronic schedule. The final injection, however, contained OXC. In all groups, the final injection was administered 30 min prior to the commencement of the seizure test. The timing of OXC administration before behavioral testing and the drug doses were determined empirically based on the greatest increase in seizure threshold. The control group and the corresponding tested group were subjected to electroconvulsions at the same time. The results from the tested groups were then compared with those of their respective controls. 

The following chemicals and reagents were utilized in the chromatographic analysis: chloramphenicol (ChF, Sigma, St. Louis, MO, USA), ethyl acetate (HPLC grade, POCH Gliwice, Gliwice, Poland), and acetonitrile (HPLC grade, POCH Gliwice, Gliwice, Poland).

### 4.3. Maximal Electroshock Seizure Test

The anticonvulsant effect of OXC was estimated in the MES test in mice. This test is a well-known animal model of tonic–clonic seizures, which is commonly used in the preclinical evaluation of anticonvulsant properties of potential antiepileptic drugs [10]. 

Electroconvulsions were produced by means of alternating current (sine wave, 0.2 stimulus duration, frequency of 50 Hz, maximum stimulation voltage of 500 V, and fixed current strength of 25 mA) delivered via ear-clip electrodes by a rodent shocker generator (Type 221, Hugo Sachs Elektronik, Freiburg, Germany). The endpoint was defined as tonic hindlimb extension, which is the state when the hindlimbs of mice are extended 180° to the plane of the body axis. A dose–response curve was calculated on the basis of the percentage of mice protected (protection in less than 50%, around 50%, and more than 50% of animals) in accordance with the methodology proposed by Litchfield and Wilcoxon [52]. Further details regarding the parameters of the generator and the methodology for calculating ED50 can be found in other sources [53]. The ED_50_ values obtained in the four protocols were designated as ED_50_1 (single-treatment protocol) and ED_50_2 (chronic protocol) for subsequent studies.

### 4.4. Chimney Test

The effects of acute and chronic treatment with OXC on motor performance in mice were determined in the chimney test [54]. The drug was administered twice daily for 14 days (i.e., in accordance with the protocol that provided the maximal total dose) at ED_50_1 and ED_50_2 doses calculated earlier. The specifics of the chimney testing procedure are delineated in the paper of Banach and Borowicz [53].

The neurotoxic effect of OXC was expressed as the median toxic dose (TD_50_) in mg/kg, which represents the dose at which OXC induced motor impairment in 50% of mice. In order to determine the median toxic dose (TD_50_), at least four groups of ten animals were injected with progressively increasing doses of the drug and subsequently challenged with the chimney test. A dose–response curve was subsequently calculated on the basis of the percentage of mice exhibiting motor deficits. The protective index was calculated by dividing the TD_50_ by the ED_50_ obtained in the acute and chronic (14 × 2) protocol.

### 4.5. Step-Through Passive Avoidance Task

The step-through passive avoidance task is regarded as a measure of long-term memory [55]. In this test, OXC was administered both acutely and chronically (in the most extensive 14 × 2 protocol) at doses of ED_50_1 and ED_50_2. The mice were placed in an illuminated box (10 × 13 × 15 cm) connected to the larger dark box (25 × 20 × 15 cm) equipped with an electric grid floor. The animal’s entrance into the dark box was met with an appropriate electric foot shock (0.6 mA for 2 s). Mice that did not enter the dark compartment within 60 s were excluded from further experimentation. On the following day (24 h later), the pre-trained mice were placed once again into the illuminated box and observed for up to 180 s. Animals that avoided the dark compartment for 180 s were considered to have retained the ability to remember the task. The control mice, which were administered a vehicle solution, did not enter the dark box within the observation period. The time elapsed between the commencement of the trial and the mice’s entry into the dark compartment was recorded, and the median latencies (retention times) with 25th and 75th percentiles were calculated.

### 4.6. Measurement of Plasma and Brain Concentrations of Oxcarbazepine

Animals were treated with OXC according to four chronic protocols. The acute protocol was used as a control group. The last dose of OXC in the chronic regimen was injected 30 min before the respective procedure (the same time as that scheduled for the electroconvulsive test). Mice were killed by decapitation. 

Blood samples were collected in Eppendorf tubes (Merck KGaA, Darmstadt, Germany) and centrifuged at 5000 rpm for 5 min. Supernatants were frozen at −80 °C. 

Brains were removed from skulls and frozen at −80 °C. After thawing, brains were weighed and homogenized with distilled water (2:1 vol/wt) by using an UltraTurrax T8 homogenizer (IKA-WERKE, Stauffen, Germany). Homogenates were centrifuged at 10,000 rpm for 10 min and the supernatants were returned to the freezer. 

Subsequently, plasma and brain homogenates were subjected to high-performance liquid chromatography (HPLC) analyses for OXC concentration determination. 

An amount of 20 µL of chloramphenicol (ChF) (8 µg/mL), as an internal standard, was added to 200 µL of serum, homogenate, and OXC solutions (Sigma) (1, 2.5, 5, 10 µg/mL). Subsequently, 2 mL of ethylacetate (HPLC-grade, POCH Gliwice) was added. Samples were mixed (vortex) for 20 s, shaken for 30 min, and centrifuged for 10 min at 4000 rpm. The upper organic phases were transferred to conical tubes and separated. The aqueous phase was re-extracted with ethyl acetate (2 mL). The organic phases were evaporated to dryness at 40 °C under a gentle stream of nitrogen. The resulting residues were reconstituted in 200 µL of acetonitrile (HPLC grade, POCH Gliwice), mixed, and injected (20 µL) into the chromatograph. The retention time of OXC was 5.3 min.

The chromatograph (Dionex, Sunnyvale, CA, USA) was equipped with a gradient pump, P580 LPG, a UV/Vis detector (UVD 340S) (Dionex, Sunnyvale, CA, USA), and a manual injector valve with a 20 µL sample loop. An analytical HPLC column, Zorbax SB C18 4.6 × 150 × 5 µm (Agilent, Santa Clara, CA, USA), was used. The mobile phase consisted of acetonitrile and 40 mM triethylammonium phosphate buffer (7:3 *v*/*v*) (Fluka, HPLC grade, Honeywell Research Chemicals, Chicago, IL, USA). The mobile phase flow rate was set as 1.0 mL/min. The detection was conducted at a wavelength of 250 nm. The concentration of OXC was determined by comparing the peak height of the OXC/ChF ratio to the peak height of the OXC to ChF calibration curve. The calibration curve exhibited linearity within the range of 0.75 to 10 µg/mL of OXC. The within-batch and between-batch precision was below 8 and 7%, respectively. The brain concentrations of OXC were expressed in µg/mL of supernatants as the means ± SD of at least eight determinations.

### 4.7. Statistics

The ED_50_ value (the dose required to protect 50% of mice against tonic convulsions) was assessed for OXC in each protocol of administration. In turn, the toxic dose in the chimney test (TD_50_) and the quotient of TD50 and ED50 (protective index) were calculated in two protocols of treatment (1 × 1 and 14 × 2). The ED_50_ and TD_50_ values were estimated using computer log-probit analysis according to Litchfield and Wilcoxon [52], with their respective 95% confidence limits. Subsequently, the standard error of the mean (SEM) was calculated. A one-way analysis of variance (ANOVA) was employed to perform multiple comparisons of the ED50 values (with their respective standard error of the mean) from the MES test. The post hoc Tukey test was then applied as a means of identifying any significant differences between the ED50 values. 

The qualitative variables derived from the chimney test were compared using the Fisher’s exact probability test. The results obtained in the step-through passive avoidance task were statistically evaluated using the Kruskal–Wallis nonparametric analysis of variance (ANOVA) followed by the post hoc Dunn test [56].

One-way analysis of variance (ANOVA) was employed to evaluate plasma and brain concentrations of OXC, with Dunnett’s post hoc test subsequently applied. The significance level was set at *p* ≤ 0.05. 

## 5. Conclusions

A two-week course of oxcarbazepine treatment was demonstrated to reduce the antielectroshock activity and the neurotoxic effects observed in mice. The obtained results are dependent on pharmacodynamic, rather than pharmacokinetic, events. The observed change in the anticonvulsant effect of OXC during chronic administration suggests that a single administration of drugs in experimental studies may not be sufficient to achieve reliable outcomes. As direct extrapolation of the effects observed in non-epileptic animals to epileptic animals is not possible, our results must be confirmed in chronic seizure models and, potentially, genetically altered mouse models of seizures.

## Figures and Tables

**Table 1:** Effects of acute and chronic treatment with oxcarbazepine (OXC) on motor performance in the chimney test.

Drug and Dose (mg/kg)	Treatment Protocol	Animals Impaired (%)
Vehicle	1 × 1	0
Vehicle	14 × 2	0
OXC 12.9 (ED_50_1)	1 × 1	0
OXC 12.9	14 × 2	0
OXC 19.9 (ED_50_2)	1 × 1	0
OXC 19.9	14 × 2	0

Data are expressed as the percentage of animals that failed to perform the chimney test. Statistical analysis of data was performed by using Fisher’s exact probability test. OXC—oxcarbazepine; treatment protocols: 1 × 1, 1 injection; 14 × 2, 2 injections daily for 14 days; ED_50_—median effective dose.

**Table 2:** Effects of acute and chronic treatment with oxcarbazepine (OXC) on its protective index in mice.

Treatment Protocol	ED_50_ (mg/kg)	TD_50_ (mg/kg)	PI
OXC 1 × 1	12.9 [11.3–14.7]	66.4 [61.8–71.3]	5.15
OXC 14 × 2	19.9 [17.4–22.9]	89.6 [78.6–101.7] ***	4.5

The data are expressed as the median effective doses (ED_50_s in mg/kg) that protected 50% of the animals against the maximal electroshock-induced seizures and the median toxic doses (TD_50_s in mg/kg) that produced motor impairment in 50% of the animals tested in the OXC chimney test, with 95% confidence limits in parentheses. The protective index (PI) is defined as the quotient of the toxic dose 50 (TD_50_) and the effective dose 50 (ED_50_) values. OXC—oxcarbazepine; treatment protocols: one injection was administered once, followed by 14 injections, two per day, for 14 days. *** *p* < 0.001 vs. OXC 1 × 1.

**Table 3:** Effects of acute and chronic treatment with oxcarbazepine (OXC) on long-term memory in mice.

Drug and Dose (mg/kg)	Treatment Protocol	Retention Time (s)
Vehicle	1 × 1	180 (180; 180)
Vehicle	14 × 2	180 (180; 180)
OXC 12.9 (ED_50_1)	1 × 1	180 (180; 180)
OXC 12.9	14 × 2	180 (91; 180)
OXC 19.9 (ED_50_2)	1 × 1	180 (180; 180)
OXC 19.9	14 × 2	180 (100; 180)

The data are expressed as median retention time (with 25th and 75th percentiles), during which the animals avoided the dark compartment in the step-through passive avoidance task. The statistical analysis of the data was conducted using the nonparametric Kruskal–Wallis ANOVA test, followed by Dunn’s post hoc test. OXC—oxcarbazepine; treatment protocols: 1 × 1, 1 injection; 14 × 2, 2 injections daily for 14 days; ED_50_—median effective dose.

## Data Availability

The data presented in this study are available in the article.
